# Longitudinal Analysis of Bone Metabolic Markers and Bone Mechanical Properties in STZ-Induced Diabetic Rats

**DOI:** 10.3390/jcm13185595

**Published:** 2024-09-20

**Authors:** Ewa Tomaszewska, Piotr Dobrowolski, Siemowit Muszyński, Janine Donaldson, Marcin Gołyński, Jowita Zwolska, Mateusz Szadkowski, Maciej Osęka, Maria Mielnik-Błaszczak, Ireneusz Balicki

**Affiliations:** 1 Department of Animal Physiology, Faculty of Veterinary Medicine, University of Life Sciences in Lublin, 20-950 Lublin, Poland; 2 Department of Functional Anatomy and Cytobiology, Institute of Biology, Maria Curie Sklodowska University, 20-033 Lublin, Poland; piotr.dobrowolski@mail.umcs.pl; 3 Department of Biophysics, Faculty of Environmental Biology, University of Life Sciences in Lublin, 20-950 Lublin, Poland; siemowit.muszynski@up.lublin.pl; 4 School of Physiology, Faculty of Health Sciences, University of the Witwatersrand, Parktown, Johannesburg 2193, South Africa; janine.donaldson@wits.ac.za; 5 Veterinary Medicine Institute, Faculty of Biological and Veterinary Sciences, Nicolaus Copernicus University in Toruń, 87-100 Toruń, Poland; marcingolynski@umk.pl; 6 Department and Clinic of Animal Surgery, Faculty of Veterinary Medicine, University of Life Sciences in Lublin, 20-950 Lublin, Poland; jowita.zwolska@gmail.com (J.Z.); matszadkowski@gmail.com (M.S.); balicki.ireneusz@gmail.com (I.B.); 7 Hospital Emergency Ward, Specialist Hospital Miedzylesie, 04-749 Warsaw, Poland; maciej_oseka@oftalabs.pl; 8 Chair and Department of Developmental Dentistry, Medical University of Lublin, 20-093 Lublin, Poland; mielnikmb@gmail.com

**Keywords:** diabetes mellitus, streptozotocin, rat, bone

## Abstract

**Background:** This longitudinal study examined the early effects of type 1 diabetes on bone mechanical properties and metabolic markers in mature rats, focusing on the natural progression of diabetes-induced changes without external treatments. **Methods**: Forty-eight 8-month-old male Wistar rats were divided into two groups, with one group receiving a single dose of streptozotocin (STZ, 60 mg/kg). Assessments were performed 2, 4, and 8 weeks post-administration, including serum biochemical analyses, bone marker assessments, and mechanical bone tests. The data were analyzed using two-way ANOVA to evaluate the impact of time and treatment. **Results**: At 2 weeks, diabetic rats showed increased fasting blood glucose (*p* < 0.001), decreased insulin levels (*p* = 0.03), and changes in HOMA markers (*p* < 0.001), liver enzymes (*p* < 0.001), inflammatory markers (*p* < 0.001), and bone metabolism markers (osteocalcin (*p* < 0.001), OPG (*p* = 0.006), RANKL (*p* < 0.001), and OPG/RANKL ratio (*p* < 0.001)), with initial alterations in bone geometry. By week 4, decreased body weight in the diabetic group (*p* < 0.001) led to further changes in bone geometry and initial differences in mechanical properties. At 8 weeks, significant declines in body (*p* < 0.001) and bone (*p* < 0.001) weights were observed, along with further deterioration in bone geometry and mechanical properties. **Conclusions**: The study highlights the significant impact of STZ-induced diabetes on bone health as early as two weeks post-STZ administration, with marked temporal changes in biochemical markers and mechanical properties.

## 1. Introduction

Diabetes mellitus (DM) is a common, multifaceted, widespread metabolic disorder considered one of the fastest-growing public health problems in the world [1]. DM is categorized into two main types: type 1 DM (T1DM) and type 2 DM (T2DM). T1DM results from the immune system attacking the pancreatic beta cells that produce insulin. It typically starts at a young age, often during childhood and adolescence. Obesity is not a usual risk factor for T1DM, and those affected are usually of normal or thin build [2]. In T2DM, the body’s cells become resistant to insulin’s effects and the pancreas cannot produce enough insulin. This type is often linked with obesity, and the two conditions frequently coexist. Additionally, obesity on its own raises the risk of developing T2DM and makes it more challenging to manage blood glucose levels [3]. Depending on the cause, DM varies in terms of significant health issues, pancreatic damage, obesity resulting from various mechanisms, and even mortality. Between the years 2000 and 2019, DM accounted for the deaths of 139,651 men and 144,398 women in America [4]. It has been predicted that the global prevalence of DM will reach up to 642 million by the year 2040 [5].

Both types of DM are linked to many side effects, including osteopenia or osteoporosis [6]. Diabetic patients might experience bone and mineral abnormalities due to various factors, including the direct impact of insulin deficiency or resistance and hyperglycemia on bone tissue. Additionally, advanced glycation end products of bone matrix proteins, irregular production of cytokines and adipokines, and their negative influence on bone cells can contribute to these issues [7]. The fundamental mechanisms involved in diabetic bone loss are well known and have been studied in different models like cell culture, human clinical observations, and rodent models [8]. Animal models serve as a middle ground between clinical trials and in vitro studies. They offer the advantage of genetic manipulation and controlled diet and environment, enabling a wide range of analyses. Thus, animal models serve as valuable tools for exploring tissue pathology mechanisms, as long as the tissue responses mirror clinical observations [9].

Streptozotocin (STZ), a nitrosurea compound originating from Streptomyces achromogenes and a cytotoxic glucose analogue [10], has served as an antibiotic and cancer treatment in the past due to its inhibition of DNA synthesis and free radical generation [11,12]. It enters pancreatic β-cells via glucose transporter 2 (GLUT2) channels within the cell membrane, inducing cellular death by DNA fragmentation and triggering local immune reactions that are not observed in humans due to a very low level of GLUT2 transporter expression [13,14]. These processes ultimately result in inflammation, endothelial dysfunction, hypoinsulinemia, and hyperglycemia in animals [15,16]. Hence, the streptozotocin-induced diabetes model has been widely employed, making it particularly beneficial for expanding and comparing outcomes across various studies [17]. This model enables the conduction of investigations involving induced T1 or T2DM, depending on a high-fat diet and whether STZ is administered once off or multiple times [9,15]. The STZ-induced diabetic rodent model has numerous advantages: it permits the induction of diabetes under controlled environmental conditions, facilitates regular monitoring and direct measurement of serum and bone factors, and provides the flexibility to select the timing of diabetic induction and the collection of bone samples. Recent observations indicate that the quality of bones, encompassing microarchitectural composition and strength characteristics, could be compromised in DM regardless of its type, potentially leading to elevated fracture susceptibility [18,19,20,21]. Although many previous animal studies have investigated general bone mechanical properties like breaking strength, energy absorption capacity, stiffness, toughness of long bones, serum biochemical parameters, and even trabecular bone using micro-computed tomography analyses [9,22,23,24,25,26], none have presented a comprehensive analysis linking bone properties with serum parameters, regulators of bone metabolism, liver toxicological parameters, and blood morphology.

Recent epidemiological data have shown that more than half of all new cases of type 1 diabetes occur in adults. It primarily results from the active development of tools that differentiate between type 1 and type 2 diabetes. Moreover, key genetic, immune, and metabolic differences exist between adult- and childhood-onset type 1 diabetes, many of which are not well understood [27]. From 2001 to 2015, youths 10–14 years old had the highest incidence of new-onset type 1 diabetes. Overall, children and adolescents 0–19 years old had a new-onset rate of 34.3 per 100,000 persons, while adults aged 20–64 had a rate of 18.6 per 100,000 persons [28]. In 2021, an estimated 8.4 million people globally had type 1 diabetes (with a 95% uncertainty interval of 8.1–8.8 million). Of these, 1.5 million (18%) were under 20 years old, 5.4 million (64%) were between 20 and 59 years old, and 1.6 million (19%) were aged 60 or older. Additionally, around 35,000 undiagnosed individuals died within 12 months of the onset of symptoms [29].

Using 8-month-old rats with uncontrolled diabetes could provide a more representative and mature model that is hypothesized to demonstrate the intricate relationship between metabolic and bone-related parameters during the early stages of DM development, potentially stemming from both the immunological and systemic impact of uncontrolled diabetes. This approach focuses on understanding the natural progression of diabetes-induced changes in bone and metabolic markers without the interference of external treatments, potentially revealing insights into both the immunological and systemic impacts of uncontrolled diabetes.

This study aims to investigate the relationship between changes in bone mechanical properties, serum biochemical parameters, regulators of bone formation and resorption, toxicological liver parameters, and basal blood morphology in rats during the early stages of STZ-induced type 1 diabetes compared to rats without diabetes.

## 2. Materials and Methods

### 2.1. Reagents

Unless specified otherwise, all chemicals were sourced from Merck KGaA, Darmstadt, Germany.

### 2.2. Animals

The study involved 48 male Wistar rats, 8 months old, with initial body weights between 350 and 450 g obtained from the Experimental Medicine Center at the Medical University of Lublin, Poland. This age group allows for the study of the effects of experimental variables on fully developed physiological systems, providing insights into conditions that may manifest or progress in adulthood and are less likely to undergo significant growth-related changes. Only male rats were used in the study since female rats are less sensitive to STZ [30]. The rats were acclimated to the laboratory conditions for 7 days before the study began. All rats were housed individually, under a 12 h light/dark cycle, a stable temperature of 22 ± 1 °C, and air exchange rates of 15–20 renewals per hour. Rats had free access to food and water. They were fed a standard rodent chow (Snif Spezialdiäten GmbH, Soest, Germany) that met the nutritional requirements of the AIN-93 diet. Fasting blood glucose (FBG) levels were measured after fasting overnight using a glucometer (AccuChek Active Performa, Roche, Germany), via the tail vein, to confirm normal fasting blood glucose levels.

### 2.3. Experimental Groups

The rats were divided into two main groups, with each group consisting of 24 rats based on whether they received streptozotocin treatment. Further, each group was subdivided into three subgroups of 8 rats each. The randomization process occurred before any experimental intervention to ensure that the groups were comparable at the start of the study. Randomization was conducted by an independent researcher who was not directly involved in the experimental procedures to minimize bias. Male Wistar rats given a single intraperitoneal injection of STZ (60 mg/kg body weight in freshly prepared 0.1 M citrate buffer) under ketamine anesthesia to induce T1DM via β-cell necrosis formed the STZ group [30]. Male Wistar rats injected with citrate buffer alone (pH 4.5), via intraperitoneal injections, formed the control group (Control group). Rats from both groups (Control, n = 8; STZ rats, n = 8) were then randomly selected, weighed, and euthanized 2, 4, and 8 weeks after STZ/buffer administration. To prevent fatal hypoglycemia caused by the destruction of beta cells and a rapid release of insulin after STZ administration, drinking water was replaced with a 10% glucose solution for 24 h, starting 6 h post-STZ treatment.

The selection of specific time points, namely 2, 4, and 8 weeks after STZ/buffer administration, is significant in understanding the dynamic progression of physiological changes associated with diabetes induction. These time points likely correspond to key stages in the development and manifestation of diabetic effects in the rat model. Two weeks: This early time point allows for the detection of initial physiological responses to STZ-induced diabetes. It is a crucial phase for observing early signs of hyperglycemia, alterations in insulin levels, and potential changes in metabolic parameters. Four weeks: By the 4-week mark, physiological changes in response to diabetes induction may have progressed and potentially stabilized or adapted. This time point provides insight into the ongoing effects of diabetes on various parameters, including metabolic, hepatic, and bone-related factors. Eight weeks: The 8-week time point represents a more chronic phase, allowing researchers to observe the long-term impact of STZ-induced diabetes. By this stage, the rats may exhibit more established patterns of hyperglycemia, potential complications, and adaptive responses in various physiological systems. Understanding the progression of diabetic effects in the rat model at these time points enhances the translational relevance of the study. It may align with the temporal patterns observed in human diabetes progression, providing insights into the applicability of the findings to clinical scenarios. At these time points, rats were fasted overnight, weighed, and anesthetized with an intraperitoneal injection of a ketamine/xylazine cocktail (100 mg + 10 mg per kg). Immediately after blood sampling for serum analysis, rats were euthanized with Euthasol vet. (400 mg/mL solution for injection, Produlab Pharma B.V., Raamsdonksveer, The Netherlands) via intracardiac injection under deep anesthesia.

### 2.4. Validation of Diabetes

Validation of diabetes was conducted 7 days after STZ injection. This allowed for a sufficient time interval to observe and confirm the sustained effects of STZ on pancreatic beta cells, ensuring the establishment of hyperglycemia. Blood glucose concentration was measured by a single needle prick on the tip of the rats’ tail, using a glucometer, in order to confirm stable hyperglycemia. This practical method is well suited for monitoring and aligns with ethical considerations. Fasting rats with blood glucose levels greater than 150 mg/dL (8.3 mmol/L) and/or statistically higher levels compared to control rats were considered diabetic [30,31].

### 2.5. Serum Assays

Blood samples were collected and allowed to clot at room temperature for 5 min before being centrifuged at 1500× *g* for 10 min at 4 °C. The serum was separated and stored at −80 °C, with no more than two freeze/thaw cycles. Serum levels of alanine transaminase (ALT), aspartate transaminase (AST), total bilirubin (TBR), urea, creatinine kinase, glucose, and total protein were analyzed using an automated biochemistry analyzer (Mindray BS-120, Bio-Medical Electronics, Shenzhen, China) with ready-to-use commercial test kits (Alfa Diagnostics, Warsaw, Poland). All analyses were verified using multiparametric control serum (Alfa Diagnostics, Warsaw, Poland). Fructosamine levels were determined using a commercial assay (F7546; Pointe Scientific Polska Sp. z o.o., Warsaw, Poland).

Serum concentrations of interleukin 1β (IL-1β, E0119Ra), interleukin 10 (IL-10, E0108Ra), and interleukin 6 (IL-6, E0135Ra) were measured using commercial rat-specific enzyme-linked immunosorbent assay (ELISA) kits from BT-Lab (Korain Biotech, Shanghai, China).

Serum insulin levels were measured using a commercial ALPCO Rat High Range Insulin ELISA kit (80-INSRTH-E01, E10, ALPCO Diagnostics Ltd., Salem, NH 03079, USA). Bone alkaline phosphatase (BALP), leptin (LEP), osteocalcin (OC), osteoprotegerin (OPG), and receptor activator of nuclear factor kappa-Β ligand (RANKL) were determined using a commercial rat-specific ELISA kit from Qayee-bio (E-EL-R1109, E-EL-R0582, E-EL-R0243, E-EL-R0050, and E-EL-R0841, Elabscience, Houston, TX, USA). All procedures followed the manufacturers’ protocols, and samples were analyzed in duplicate using a microplate spectrophotometer (Benchmark Plus, Bio-Rad Laboratories, Inc., Hercules, CA, USA). Results were calculated based on standard curves generated for each test. The OPG/RANKL ratio was calculated.

Terminal blood collection was performed after overnight fasting for the determination of the Homeostatic Model Assessment (HOMA) score for β-cell function (HOMA-β), which was calculated using the following formula [32,33]:

(1) $$HOMA–\beta =\frac{\left(20\times insulin\right)}{\left(glucose-3.5\right)},$$

with conversion factors for insulin (1 ng/mL = 24.8 μIU/mL) and blood glucose (1 mmol/L = 18 mg/dL) [32,34,35].

### 2.6. Bone Collection

Immediately after euthanasia, the right femora of the rats were isolated and cleaned, and their weights and lengths were measured. The femora were then stored at −25 °C for further analysis. The relative bone weight and Seedor index (an indirect indicator of bone density) were calculated as the ratio of bone weight to body weight and bone weight to bone length index, respectively.

The mechanical properties of the bone were assessed through a three-point bending test using a Zwick Z010 universal testing machine (Zwick GmbH & Co. KG, Ulm, Germany). The femora were placed on supports spaced at 40% of the total bone length and the bone mid-diaphysis was loaded at a rate of 10 mm/min until fracture.

Based on the load–displacement curves, using the Origin software (ver. 2022, OriginLab, Northampton, MA, USA), the following mechanical parameters were determined: yield load F_y_, ultimate load F_max_, elastic energy W_y_, work to fracture W_max_, and stiffness S [36,37]. Following the three-point bending test, the transverse (V, v) and anteroposterior (H, h) diameters (outer and inner, respectively) were measured using an electronic caliper (IP67, INSIZE, Suzhou New District, China), enabling the calculation of the following geometrical parameters of the femur mid-diaphysis: mean relative wall thickness (MRWT), cortical cross-sectional area, and cortical index [37]. Finally, Young’s modulus was calculated based on the data obtained from the three-point bending tests and the measured mid-diaphysis geometric parameters [38].

### 2.7. Statistical Analysis

Data are presented as mean ± standard error of the mean (SEM). Prior to conducting a two-way ANOVA, assumptions of normality and homogeneity of variances were verified to ensure the appropriateness of parametric testing. Normality of data was assessed using the Shapiro–Wilk test for each group at every time point. The homogeneity of variances across groups and time points was checked using the Brown–Forsythe and Bartlett’s tests. In instances where the assumptions were not met, data transformations were considered to satisfy these criteria. The study involved multiple time points and distinct groups, with individual rats serving as the experimental units. To evaluate the impact of time and treatment groups on bone metabolic markers and mechanical properties, a two-way analysis of variance (ANOVA) was conducted to assess the main effects of ‘time’ and ‘group’ on the dependent variables, as well as their interaction effect (time × group) followed by post-hoc pairwise comparisons using Tukey’s Honestly Significant Difference (HSD) test. However, this model accounts for the complexity and specificity of biological data by considering only the interactions that provide useful insights. It compares cell means within each row and column to examine the temporal and treatment-related effects most relevant to the research questions. By narrowing the analysis to biologically relevant interactions, the findings of the model become clearer and closer to the biological phenomena being studied. The model was designed to analyze interactions between ‘time’ and ‘group’ factors that are relevant to the progression of diabetes over time and in response to interventions such as changes in bone metabolic markers and mechanical properties. Given the refined approach and focus on biologically relevant interactions for analyzing the effects of STZ-induced diabetes on bone health, the mathematical model for the analysis can be described as follows: *Y_ijk_* represents the dependent variable measured (e.g., a bone metabolic marker or a mechanical property of bone) for the *i*th group (treatment vs. control) at the *j*th time point for the kth specimen. A mathematical model incorporating biologically relevant interactions has been formulated as follows:

(2) $$Y_{ijk}=\mu +\alpha_{i}+\beta_{j}+{\left(\alpha \beta \right)}_{ij}+\epsilon_{ijk},$$

where *μ* is the overall mean response of the dependent variable across all groups and time points; *αi* is the effect of the I group (treatment vs. control) on the dependent variable; *β_j_* is the effect of the *j*th time point on the dependent variable; and (*αβ*)*_ij_* is the interaction effect between the *i*th group and *j*th time point on the dependent variable, focusing only on interactions deemed biologically relevant. This captures how the effect of treatment varies over time and is critical for understanding the dynamics of STZ-induced diabetes on bone health. For nonrelevant interactions, this term is considered to be zero. *ϵ_ijk_* is the random error associated with the *k*th specimen’s measurement. This analysis allowed us to discern whether the influence of STZ-induced diabetes on bone health varied over the study duration and to compare these effects between the treated and control groups. GraphPad Prism version 10.0.2 for Windows (GraphPad Software, San Diego, CA, USA) was used for all statistical analyses, with a predetermined significance level of α = 0.05.

## 3. Results

### 3.1. Streptozotocin Effect, Basal Blood Glucose, Plasma Insulin, and Fructosamine Levels

**Figure 1:**
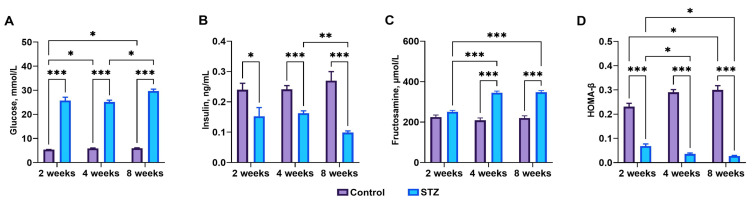
Blood serum concentrations of (**A**) glucose, (**B**) insulin, (**C**) fructosamine, and HOMA scores for (**D**) β-cell function, HOMA-β. Control—control rats, STZ—rats receiving a single i.p. injection of STZ (60 mg/kg bw) at week 0. Significance values were calculated using a two-way analysis of variance (ANOVA): * *p*-value < 0.05, ** *p*-value < 0.01, *** *p*-value < 0.001.

Control rats exhibited normal fasting serum glucose levels, while STZ-treated rats showed a significant increase in fasting blood glucose (*p* < 0.001 at all time points) along with a significant decrease in insulin concentration 2 (*p* = 0.03), 4 (*p* < 0.001), and 8 (*p* < 0.001) weeks post-STZ administration (Figure 1A). Insulin concentrations 4 weeks post-STZ administration were significantly higher than those observed at week 8 (*p* = 0.004) (Figure 1B). Fructosamine concentrations were significantly elevated at weeks 4 and 8 in STZ rats compared to those observed in STZ rats at week 2 (*p* < 0.001) and in control rats at weeks 2 (*p* < 0.001), 4 (*p* < 0.001), and 8 (*p* < 0.001) (Figure 1C).

### 3.2. Indices of Insulin Sensitivity

HOMA-β, an indicator of β-cell function, decreased gradually in STZ rats (*p* < 0.001) and was significantly lower at weeks 4 (*p* = 0.04) and 8 (*p* = 0.01) compared to week 2 post-STZ administration (Figure 1D).

### 3.3. Blood Basal Parameters

**Figure 2:**
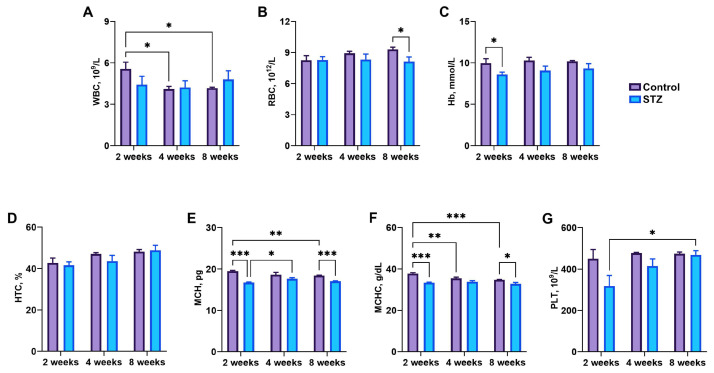
Blood hematology: (**A**) total white blood cell (WBC) numbers, (**B**) red blood cell (RBC) numbers, (**C**) hemoglobin (Hb) concentrations, (**D**) hematocrit (HTC), (**E**) mean corpuscular hemoglobin (MCH), (**F**) mean corpuscular hemoglobin concentration (MCHC), (**G**) platelet (PLT) numbers. Control—control rats, STZ—rats receiving a single i.p. injection of STZ (60 mg/kg bw) at week 0. Significance values were calculated using a two-way analysis of variance (ANOVA): * *p*-value < 0.05, ** *p*-value < 0.01, *** *p*-value < 0.001.

WBCs decreased in control rats over time (*p* = 0.02) (Figure 2A), and RBCs were reduced in STZ rats at week 8 (*p* = 0.04) compared to that observed in control rats (Figure 2B). Hemoglobin concentrations were reduced in STZ rats at week 2 compared to control rats at week 2 (*p* = 0.03) (Figure 2C). No changes in HTC were noted (Figure 2D). MCH in STZ rats at week 2 was significantly lower compared to that in control rats at week 2 (*p* < 0.001) and STZ rats at week 4 (*p* = 0.04) (Figure 2E). MCH was also reduced in STZ rats compared to that in control rats at week 8 (*p* < 0.001). MCHC gradually decreased over time in control rats (*p* = 0.004 at the 4th week and *p* < 0.001 at the 8th week), and was significantly reduced in STZ rats compared to control rats at weeks 2 (*p* < 0.001) and 8 (*p* < 0.02) (Figure 2F). The number of platelets was significantly higher in STZ rats at week 8 (*p* = 0.02) compared to that observed at week 2 (Figure 2G).

### 3.4. Basal Biochemical Analysis: IL-1β, IL-6, and IL-10

**Figure 3:**
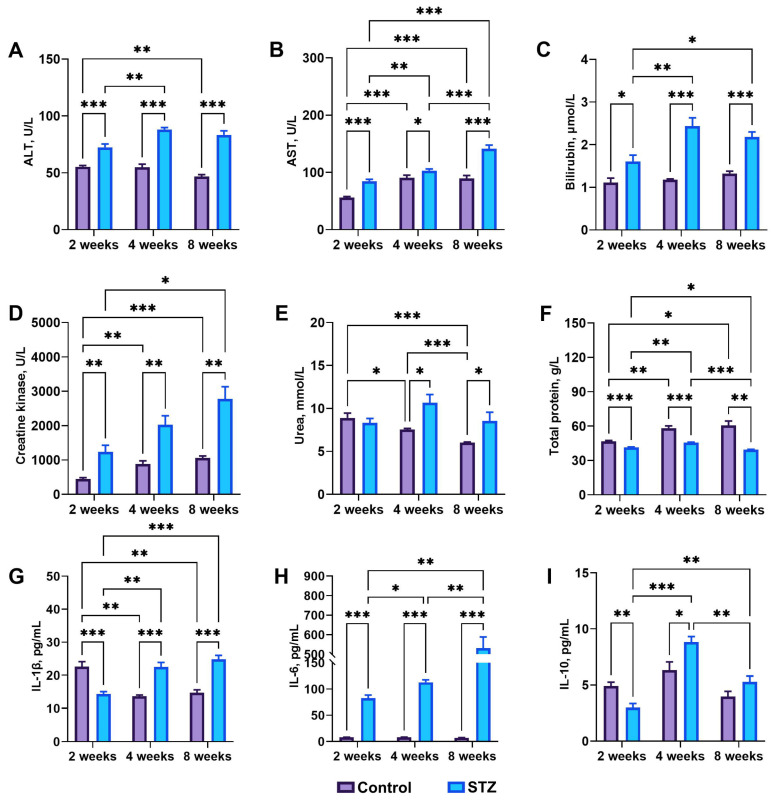
Blood serum biochemical parameters: (**A**) alanine transaminase (ALT), (**B**) aspartate transaminase (AST), (**C**) total bilirubin, (**D**) creatine kinase, (**E**) urea, (**F**) total protein, (**G**) interleukin 1β (IL-1β), (**H**) interleukin 6 (IL-6), (**I**) interleukin 10 (IL-10). Control—control rats, STZ—rats receiving a single i.p. injection of STZ (60 mg/kg bw) at week 0. Significance values were calculated using a two-way analysis of variance (ANOVA): * *p*-value < 0.05, ** *p*-value < 0.01, *** *p*-value < 0.001.

ALT (*p* < 0.001) and AST (*p* < 0.001 at the 2nd week, *p* = 0.04 at the 4th week, and *p* < 0.001 at the 8th week) activities increased significantly in all STZ rats compared to those noted in control rats (Figure 3A,B). Moreover, ALT activity in STZ rats at week 4 was higher than that observed at week 2 (*p* = 0.002). AST activity was significantly higher at weeks 4 (*p* = 0.006) and 8 (*p* < 0.001) compared to that at week 2 in STZ and control rats (*p* < 0.001 and *p* = 0.001, respectively). In general, total bilirubin was significantly higher in STZ rats compared to control rats (*p* = 0.02 at the 2nd week, and *p* < 0.001 at the 4th and 8th weeks) (Figure 3C). Moreover, total bilirubin was significantly increased at week 4 (*p* = 0.005) and decreased at week 8 (*p* = 0.04) in STZ rats compared to week 2. Creatine kinase activity, used as an indicator of muscle state, was significantly higher in all STZ rats compared to that in control rats (*p* = 0.004, *p* = 0.003, and *p* = 0.002 at weeks 2, 4, and 8, respectively), and additionally, it was significantly higher at week 8 than at week 2 in STZ rats (*p* = 0.03), and higher at week 4 than at week 2 in control rats (*p* = 0.002) (Figure 3D). Urea concentration at week 8 was significantly lower than at weeks 2 (*p* < 0.001) and 4 (*p* < 0.001) in control rats, and its activity in STZ rats was significantly higher than in control rats at weeks 4 (*p* = 0.01) and 8 (*p* = 0.04) (Figure 3E). Total protein concentrations at weeks 4 (*p* = 0.006) and 8 (*p* = 0.04) were significantly higher than that at week 2 in control rats, while it was increased at week 4 (*p* = 0.002) and decreased at week 8 (*p* = 0.02) in STZ rats compared to that observed at week 2 (Figure 3F). Moreover, total protein was significantly lower in STZ rats compared to that in control rats (*p* < 0.001, *p* < 0.001, and *p* = 0.001 in respective weeks).

IL-1β concentrations decreased at weeks 4 (*p* = 0.002) and 8 (*p* = 0.008) compared to week 2 in control rats, while in STZ rats, an increase was observed (*p* = 0.004 and *p* < 0.001, respectively) (Figure 3G). Moreover, IL-1β concentration was lower at week 2 (*p* < 0.001) and higher at weeks 4 (*p* < 0.001) and 8 (*p* < 0.001) in STZ rats compared to control rats. IL-6 concentration increased in STZ rats over time and was significantly higher than in control rats (*p* < 0.001 for all time points) (Figure 3H). IL-10 concentration was lower in STZ rats than in control rats at week 2 (*p* = 0.002), and higher in STZ rats at week 4 (*p* = 0.02). Moreover, IL-10 concentration in STZ rats was highest in the 4th week compared to the 2nd and 8th weeks (*p* < 0.001 and *p* = 0.006, respectively) (Figure 3I).

### 3.5. Body Weight and Bone Morphology

**Figure 4:**
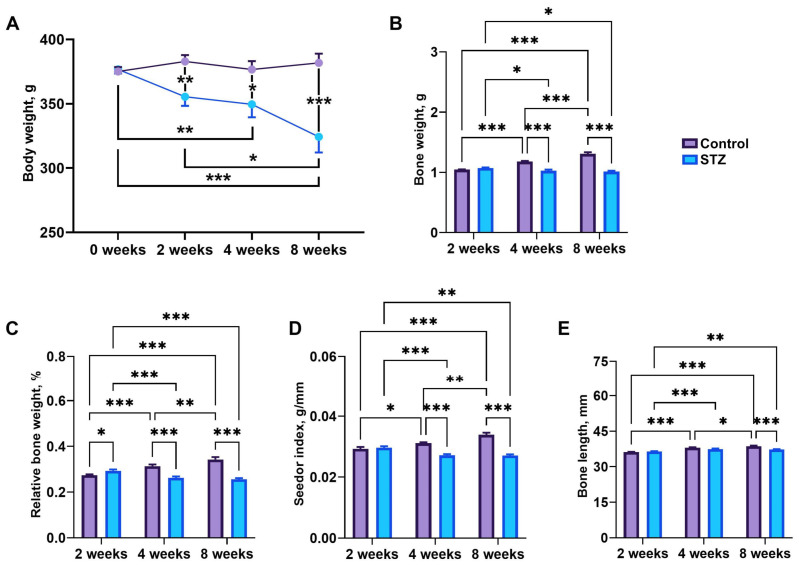
Changes in (**A**) rats’ body weight and basal femur indices. (**B**) Bone weight, (**C**) relative bone weight, (**D**) the Seedor index, (**E**) bone length. Control—control rats, STZ—rats receiving a single i.p. injection of STZ (60 mg/kg bw) at week 0. Significance values were calculated using a two-way analysis of variance (ANOVA): * *p*-value < 0.05, ** *p*-value < 0.01, *** *p*-value < 0.001.

Initial body weights were not different between groups. Body weight decreased in STZ rats compared to control rats at weeks 2 (*p* = 0.01) and 8 (*p* < 0.001), and was significantly lower at week 8 compared to that observed at week 2 (*p* = 0.03) in STZ rats (Figure 4A). Bone weight increased in control rats over time (*p* < 0.001), while a decrease in bone weight was noted in STZ rats at weeks 4 and 8, which was significant compared to that noted at week 2 (*p* = 0.03) in STZ rats (Figure 4B). Moreover, bone weight was significantly reduced in STZ rats compared to that noted in control rats at weeks 4 (*p* < 0.001) and 8 (*p* < 0.001). Relative bone weight and Seedor index increased in control rats and decreased in STZ rats over time (Figure 4C,D). Relative bone weight and Seedor index were significantly decreased at the 4th and 8th weeks in STZ groups compared to control rats (*p* < 0.001) and were decreased at weeks 4 and 8 compared to week 2 in STZ rats (*p* < 0.001). Bone length in control rats increased gradually over time (*p* < 0.001), while significantly longer bones were observed in STZ rats at weeks 4 (*p* < 0.001) and 8 (*p* = 0.003) compared to that at week 2 (Figure 4E). Moreover, STZ rats had shorter bones compared to control rats at week 8 (*p* < 0.001).

### 3.6. Bone Geometry and Mechanical Parameters

**Figure 5:**
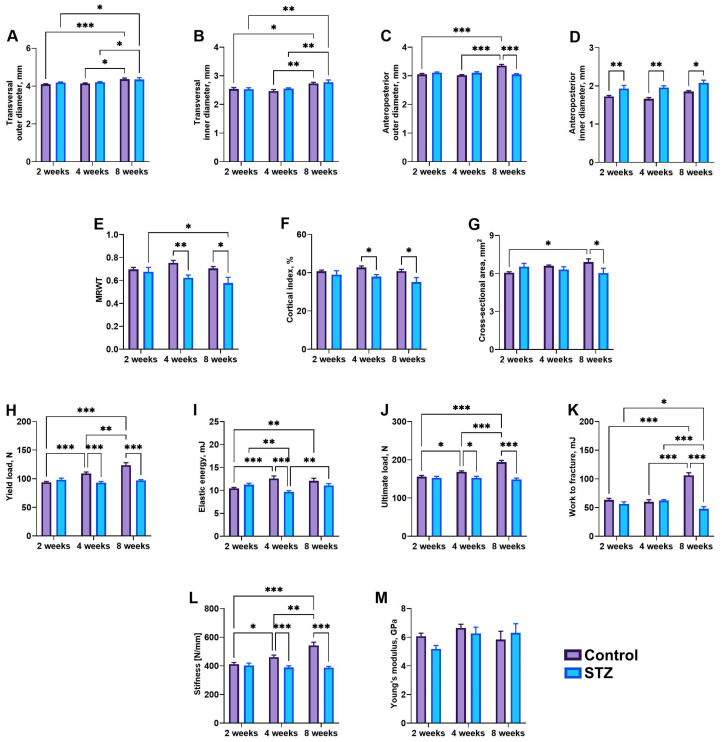
Bone geometry (**A**–**G**) and mechanical properties (**H**–**M**): (**A**) transversal outer diameter, (**B**) transversal inner diameter, (**C**) anteroposterior outer diameter, (**D**) anteroposterior inner diameter, (**E**) mean relative wall thickness (MRWT), (**F**) cortical index, (**G**) cross-sectional area, (**H**) yield load, (**I**) elastic energy, (**J**) ultimate load, (**K**) work to fracture, (**L**) stiffness, (**M**) Young’s modulus. Control—control rats, STZ—rats receiving a single i.p. injection of STZ (60 mg/kg bw) at week 0. Significance values were calculated using a two-way analysis of variance (ANOVA): * *p*-value < 0.05, ** *p*-value < 0.01, *** *p*-value < 0.001.

Transversal outer diameter increased at week 8 compared to weeks 2 (*p* < 0.001 and *p* = 0.03 for control and STZ, respectively) and 4 (*p* = 0.04 and *p* = 0.03 for control and STZ, respectively) (Figure 5A). The transversal inner diameter was significantly increased at week 8 compared to week 2 in control rats (*p* = 0.02) and was reduced in STZ rats compared to control rats at week 8 (*p* = 0.04) (Figure 5B). The anteroposterior outer diameter was increased at week 8 compared to weeks 2 (*p* < 0.001) and 4 (*p* < 0.001) in control rats, while it was decreased in STZ rats compared to control rats at week 8 (*p* < 0.001) (Figure 5C). The anteroposterior inner diameter was greater in STZ rats compared to control rats, regardless of time (*p* = 0.005 at 2 weeks, *p* = 0.001 at 4 weeks, and *p* = 0.02 at 8 weeks) (Figure 5D). Mean relative wall thickness was significantly decreased in STZ rats compared to control rats at weeks 4 (*p* = 0.01) and 8 (*p* = 0.02) (Figure 5E). The cortical index was reduced in STZ rats at weeks 4 (*p* = 0.04) and 8 (*p* = 0.02) compared to control rats (Figure 5F), while the cross-sectional area was increased at week 8 compared to that observed at weeks 2 (*p* = 0.03) in control rats and was reduced in STZ rats compared to that in control rats at week 8 (*p* = 0.03) (Figure 5G).

Yield load increased gradually in control rats (*p* < 0.001), while a decrease in yield load was noted at weeks 4 and 8 in the STZ group compared to that observed in respective controls (*p* < 0.001) (Figure 5H). Elastic energy was significantly higher in control rats at weeks 4 (*p* < 0.001) and 8 (*p* = 0.007) compared to week 2, while it was reduced in STZ rats at week 4 compared to week 2 (*p* = 0.001) (Figure 5I). Moreover, elastic energy was significantly reduced in STZ rats at week 4 compared to that in control rats (*p* < 0.001) and in STZ rats at week 8 (*p* = 0.007). Ultimate load increased gradually in control rats over time, while a reduction in ultimate load was observed in STZ rats compared to control rats at weeks 4 (*p* = 0.01) and 8 (*p* < 0.001) (Figure 5J). Work to fracture was greater at week 8 compared to weeks 2 (*p* < 0.001) and 4 (*p* < 0.001) in control rats, while it was decreased at week 8 compared to week 4 in STZ rats (*p* = 0.002) and week 8 in control rats (*p* < 0.001) (Figure 5K). Stiffness increased gradually in control rats over time, while in STZ rats, stiffness was significantly reduced compared to control rats at week 8 (*p* < 0.001) (Figure 5L). No changes in Young’s modulus were observed (Figure 5M).

### 3.7. Bone Metabolism Markers

**Figure 6:**
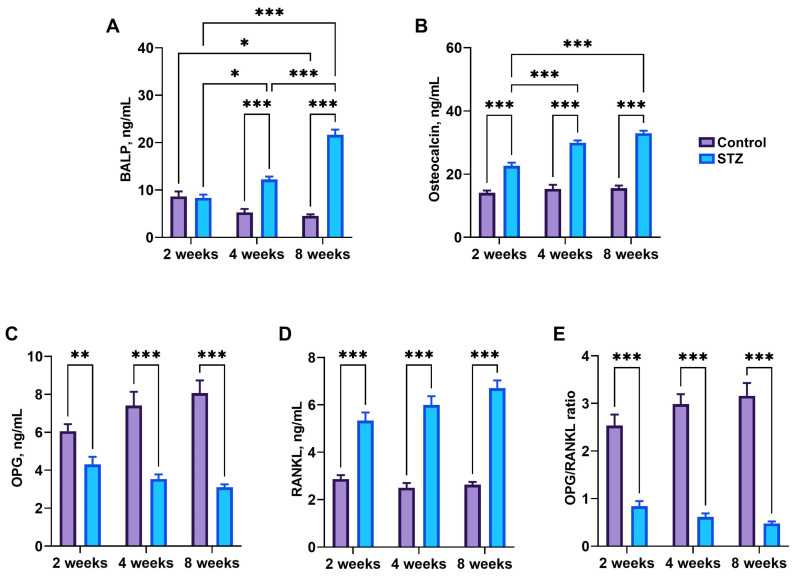
Bone metabolism markers: (**A**) bone alkaline phosphatase (BALP), (**B**) osteocalcin (**C**) osteoprotegerin (OPG), (**D**) receptor activator for nuclear factor kappa-B ligand (RANKL), (**E**) the OPG/RANKL ratio. Control—control rats, STZ—rats receiving a single i.p. injection of STZ (60 mg/kg bw) at week 0. Significance values were calculated using a two-way analysis of variance (ANOVA): * *p*-value < 0.05, ** *p*-value < 0.01, *** *p*-value < 0.001.

BALP concentration was decreased at week 8 compared to that observed at week 2 (*p* = 0.02) in control rats and increased in STZ rats at weeks 4 (*p* = 0.02) and 8 (*p* < 0.001) compared to that observed at week 2 (Figure 6A). Moreover, BALP concentration in STZ rats was significantly higher than in control rats at weeks 4 (*p* < 0.001) and 8 (*p* < 0.001). Osteocalcin concentration was significantly higher in STZ rats than in control rats (*p* < 0.001), and increased over time in STZ rats (*p* < 0.001) (Figure 6B). OPG concentration was significantly reduced (*p* = 0.006 at week 2, *p* < 0.001 at week 4, and *p* < 0.001 at week 8) (Figure 6C), while RANKL concentration was significantly increased in all STZ rats compared to control rats (*p* < 0.001) (Figure 6D). As a result, the OPG/RANKL ratio was significantly higher in STZ rats than in control rats in all examined time points (*p* < 0.001) (Figure 6E).

## 4. Discussion

Despite its clinical significance, research on the influence of adult-onset diabetes on bone tissue is still in its infancy. Older rats were specifically chosen to assess how pre-existing bone structure and metabolism respond to the onset of diabetes, which might differ significantly from younger, still-developing animals. While this does limit the study’s direct applicability to juvenile diabetes, it provides valuable insights into the chronic effects of diabetes on bone health, which are relevant for understanding the disease’s impact on adults who have had diabetes for several years.

The present study showed that the administration of STZ in a rat model leads to necrosis of pancreatic β-cells, as confirmed by HOMA-β, leading to weight loss. Selection of the experimental points at 2, 4, and 8 weeks after STZ administration corresponds to the typical timeframe during which metabolic changes associated with STZ exposure are observed [39]. This study provides a thorough examination of metabolic parameters, including glucose levels, insulin sensitivity, and markers of inflammation, offering a comprehensive understanding of the systemic effects of STZ-induced diabetes. The inclusion of multiple time points allows for a dynamic assessment of these metabolic changes over the course of 8 weeks. The current data show that STZ-induced T1DM in rats resulted in significantly higher glucose and fructosamine but lower insulin concentrations in STZ rats compared to controls, which is in agreement with previous data [40]. Kaikini et al. [41] also showed a consistently elevated level of glucose in STZ rats throughout the entire 4-week duration of the experiment. Fructosamine, a marker of glucose control reflecting the average glycemic level over the preceding 2–3 weeks [42], increased in the current study at week 4 in STZ rats. Brossaud et al. [43], however, noted an increase in fructosamine levels in rats 3 weeks following STZ administration. Changes in red blood cell indices noted in the current study were in line with previous observations, indicating an initial increase in RBC and a decrease in long-lasting diabetes [44]. Data presented here also confirm that diabetes affects the morphological structure of erythrocytes [45]. Elevated creatine kinase could be an indication of protein catabolism in skeletal muscles, consistent with the reduced body weight and increased total protein and urea concentration observed in STZ rats, which is also in line with other studies [46,47]. Reduced body weight caused by a decrease in muscle mass was also confirmed by Motyl and McCabe [9] in mice. Previous data have also suggested liver injury following STZ administration; however, Leeds et al. [48] have maintained that increased ALT and AST activities are uncommon in type 1 DM. On the other hand, Stadler et al. [49] affirmed that elevated liver enzymes are associated with worsened glycemic control; this was also confirmed by Yazdi et al. [50], who revealed that it is a result of liver damage caused by hyperglycemia-induced oxidative stress and disturbed liver metabolism, which has also been observed in other experimental diabetic models. It can be assumed that the increase in bilirubin observed in STZ rats in the present study is a consequence of metabolic disturbances. Udo et al. [51] presented increased bilirubin levels in diabetic rats 3 weeks after STZ administration. High bilirubin levels serve as a powerful endogenous anti-oxidant and anti-inflammatory agent [52], and may be protective against the autoimmune inflammation-related pathology of type 1 diabetes [53]. On the other hand, the increase in bilirubin could be caused by the shortened lifespan of RBCs, the number of which was significantly reduced in STZ rats at week 8, which is contrary to data presented by Scridon et al. [54] who showed unchanged RBC numbers in STZ-induced diabetic rats even at week 38.

The STZ-induced diabetes presented in this study was associated with increased IL-1β levels, contributing to inflammation and β-cell destruction. IL-6 in STZ-induced diabetes may exhibit both proinflammatory and immunoregulatory actions. It can contribute to inflammation, but it might also play a role in modulating the immune response in the context of STZ toxicity. While elevated from the 4th week, IL-10 could help counterbalance the proinflammatory effects associated with STZ and limit the severity of inflammation. It is important to recognize that while STZ-induced diabetes shares some similarities with autoimmune T1D, there are distinct differences in the underlying mechanisms. STZ directly damages β-cells, leading to a condition that resembles T1D but lacks the autoimmune component seen in natural T1D. The immune response in STZ-induced diabetes is more focused on the consequences of β-cell toxicity, rather than an autoimmune attack [55]. IL-1β and IL-6 are also implicated in bone resorption, while IL-10 could potentially counteract the bone-resorptive effects of proinflammatory cytokines. In conditions associated with chronic inflammation, such as autoimmune diseases or certain metabolic disorders like T1DM, the balance between these cytokines can be disrupted, leading to increased bone loss, like that observed in the current study as soon as 4 weeks after STZ administration. Similar data are presented by Gennaro et al. [56], who also showed a decrease in OPG-positive cells in the bone on day 60 and an increase in RANKL-positive cells at week 2 post STZ treatment. In the current study, the serum OPG concentration decreased gradually from the 2nd week after STZ administration, with a simultaneous increase in RANKL, which resulted in a decrease in the OPG/RANKL ratio. Zheng et al. [57] observed a decrease in OPG and the OPG/RANKL ratio and an increase in osteocalcin and alkaline phosphatase activity 8 weeks post-STZ treatment. In the current study, BALP activity increased gradually from week 4 after STZ administration. The current study also showed an increase in leptin at week 4, followed by a decrease at week 8. There is a paucity of data relating to the effect of STZ on leptin levels. One study by Gülen and Dinçer [58] presented a rapid decrease in leptin concentrations in rats 2 weeks after STZ administration, which is in contrast to data from the current data. The authors concluded that STZ-treated rats, often used as a study model of T1DM, are characterized not only by decreased insulin levels and hyperglycemia, but also by hypoleptinemia. This difference could be a result of the different initial weights of the rats used in these two studies. The current study involved rats weighing about 450 g, but Gülen and Dinçer [58] used much younger rats with a body weight of about 250 g. It should be mentioned that leptin is predominantly expressed by adipocytes, and its plasma levels correlate well with body fat mass (which one can assume is greater in heavier rats). The observed leptin patterns, differing from previous findings, underscore the importance of considering factors such as initial body weight and composition in interpreting leptin responses in diabetic models. On the other hand, bone loss accompanied by decreased markers of osteoblast activity is common in T1 diabetic patients and animal models [59,60,61]. Although STZ rats exhibited reduced body weights as early as week 2, the manifestation of bone loss was observed by week 4. During this period, the femur showed a decrease in length and density compared to control rats (the growth is still noted in rats albeit at a slower rate than in younger rats). In the study by Waud et al. [62], where 80-day-old BB/Wor rats were used as an accepted model for human autoimmune insulin-dependent T1DM, no decrease in femoral BMD was observed at weeks 3, 6, and 9. A decrease was noted only at week 12, but without changes in plasma osteocalcin. On the other hand, Motyl and McCabe [9] demonstrated bone loss in STZ mice on day 19, which was intensified on day 40 without any changes in bone growth—confirmed by the lack of differences in tibial length—although 10-week-old mice still exhibited skeletal growth. Waud et al. [62] suggested that the decrease in BMD observed in rats that spontaneously developed diabetes is related to the diabetic state only and not the immune abnormalities responsible for diabetes in this animal model, and that in STZ diabetic rats, osteoporosis develops due to inhibited fibroblast activity, which alters collagen production. The authors postulated the following pathogenetic mechanism for bone loss in diabetic rats: increased urinary calcium excretion, reduced levels of parathyroid hormone, decreased intestinal calcium absorption, altered vitamin D metabolism, increased adrenal gland function, abnormal collagen metabolism, and primary dysfunction of the osteoblast [62]. The current study identified early changes in proinflammatory cytokine (IL-1β and IL-6) and anti-inflammatory cytokine (IL-10) levels. This early cytokine imbalance may contribute to the observed bone changes, providing insights into the intricate interplay between inflammation and bone metabolism in the context of diabetes. Data presented in the current study also indicated that decreased osteoblast recruitment and/or function, and increased osteoclast function were responsible for the bone loss, as confirmed by the bone turnover markers. However, Motyl and McCabe [9] noted the loss of trabecular bone. Although the loss of trabeculae was not presented in the current study, the values of all diameters indicate thinning of the femoral midshaft, consisting of compact bone, which was supported by a reduction in other geometric parameters. Horcajada-Molteni et al. [63] observed no alterations in the femoral diameter in STZ rats after 40 days; however, they calculated it as the mean of the greatest and the smallest femoral diaphysis diameters. In another study involving 12-week-old STZ diabetic rats weighing 210–300 g, assessment on week 8 revealed reduced parameters of mechanical testing: bone cross-section [64,65]. This is consistent with the changes detected in the current study, such as definitive shortening of the femur, reduced cross-sectional area, and reduced bone strength, which, however, appeared earlier at weeks 2 and 4 after administration. On the other hand, these results are in agreement with clinical findings showing that the tibial radius of T1DM adolescent boys is smaller than that of diabetic girls [66]. The authors of that study suggested that abnormalities in bone biomechanics are direct effects of insulin deficiency and hyperglycemia on the bone and advanced glycation end products of bone matrix proteins, abnormal cytokine and adipokine production, and their detrimental effects on bone cells [66]. Collagen cross-links are divided into two types: enzymatic and glycation or oxidation-induced AGEs. An increase in AGE formation in the bones of diabetics results in a reduction in bone strength. Although the current study is limited by the lack of trabecular histomorphometry, it demonstrates that bone changes, including decreased length and density, begin as early as 4 weeks after streptozotocin (STZ) administration. However, the first visible changes in bone geometry, such as widening of the medullary cavity, are observed as early as 2 weeks. This rapid manifestation of bone loss highlights the dynamic nature of skeletal changes in response to uncontrolled diabetes, distinguishing it from other studies that observed bone alterations at later time points or assessed bones at only one time point. Unlike studies focusing on trabecular bone, this investigation explores changes in femoral midshaft geometry, emphasizing alterations in inner and outer diameters. The observed increase in internal diameter in STZ rats suggests a potential imbalance between endosteal resorption and periosteal apposition, influencing bone mechanics.

## 5. Conclusions

In conclusion, the current study investigated the impact of streptozotocin (STZ) treatment on various physiological and biochemical parameters in rats, with a specific focus on bone metabolism. STZ-induced diabetic rats exhibited altered glucose homeostasis, insulin resistance, and changes in blood parameters, indicating systemic effects. Moreover, the study revealed significant alterations in bone morphology, geometry, and mechanical parameters in STZ-treated rats compared to controls. These changes included reduced bone weight, relative bone weight, the Seedor index, and alterations in bone length and diameter. Additionally, bone metabolism markers, such as BALP, osteocalcin, OPG, RANKL, and the OPG/RANKL ratio, were significantly different between STZ and control groups. Overall, the findings suggest that STZ-induced diabetes has systemic effects, impacting bone health and metabolism in rats. The timely emergence of these findings at 2 weeks post STZ administration underscores the rapid and dynamic nature of the physiological changes induced by STZ treatment. These insights contribute valuable information about the early onset of systemic and skeletal consequences of diabetes, offering a nuanced understanding of the intricate interplay between metabolic disorders and bone physiology. The study hints at the importance of the duration of uncontrolled hyperglycemia, suggesting that short-term effects on bone properties may be subtle or undetectable, while longer-term exposure could lead to more pronounced skeletal consequences. This temporal perspective enhances understanding of the time-dependent impact of diabetes on bone health.

## Data Availability

The data presented in this study are available on request from the corresponding author.
